# Topics of the Month

**Published:** 1894-06

**Authors:** 


### TOPICS OF THE MONTH.

THE Women's Medical Club of Buffalo held their banquet at the Hotel Niagara, Monday evening, April 30, 1894, which was, as usual, well attended. Mrs. Cora Billings Lattin, the president, acted as toastmaster. The toasts were responded to by Dr. Maud J. Frye, who spoke to Women as Clinicians; Mrs. Amelia E. Trant to The Quiz Masters; Dr. Ida C. Bender to The Physician to Reform Education; Dr. N. Victoria Chappell, The Evolutions of Practice; Dr. Jane W. Carroll responded to The Microbe; Dr. Frear, Our Returned Surgeon; Dr. Electa B. Whipple, The Specialist; Dr. Lillian Craig Randall, The Mother as a Medical Woman; while Miss Westlake responded to The Boys, and Miss Ella M. Braids answered to The Graduates. The speeches were bright, witty and entertaining. These are among the most enjoyable dinners of the doctors' festive season.

The Craig colony for epileptics to be established at Sonyea, Livingston county, is a step forward in a direction that has long been needed in this State. The bill passed by the legislature, now become a law, appropriates $140,000 for this purpose. It is a significant fact that it received but four negative votes in the assembly and passed the senate unanimously.

For some time past, alienists, neurologists and philanthropists have been agreed as to the fact that epileptics should be removed from the county poorhouses and grouped in colonies specially prepared to meet their necessities for employment, education and medical treatment. Such colonies have long existed in foreign countries, but the first State institution for epileptics in this country was founded in Ohio in 1891. Many other states are now moving to accomplish the same purpose. The 600 or 700 epileptics now scattered throughout the State will be grouped in this colony, which is to be established on the cottage plan. The institution will be named after the late Oscar Craig, of Rochester, who, as president of the State Board of Charities, was instrumental in its establishment.

Governor Flower has appointed the following named board of managers: Dr. Frederick Peterson, New York City; Mrs. C. F. Wadsworth, Geneseo; George M. Shull, Mount Morris; Dr. C. E. Jones (homeopath), Albany; W. H. Cuddeback, Buffalo. The board has organized by the selection of Dr. Peterson as president, and Mr. Shull as secretary. The managers serve without salary and meet at the colony once a month, or oftener, though it is not expected to be in readiness to receive patients before the Autumn of 1895.

---

The Post-Graduate Medical School and Hospital formally opened its new building at Second avenue and Twentieth street, New York, during its jubilee week, beginning April 23, 1894. This handsome structure is admirably adapted to the purposes of post-graduate instruction, which, of course, is entirely of a clinical nature. During the twelve years that this institution has been in operation, its success has been almost phenomenal. The projectors and the supporters of the scheme, together with the present faculty, are to be congratulated upon occupying this model home for teaching and treating disease. To the distinguished president of the Post-Graduate, Dr. D. B. St. John Roosa, the Journal particularly desires to extend its congratulations at this time upon the fruition of his long-cherished hopes.

Dr. George F. Shrady, editor of the *Medical Record*, through the columns of that journal, has raised over $8,000 for a statue to the memory of Dr. J. Marion Sims, the founder of gynecology as a separate branch of medicine, and of the first Woman's Hospital in the world. The statue, which is now in the custody of a safe deposit company, was made by DuBois, the great French sculptor. It is a figure of heroic size, seven and one-half feet from the plinth, cast in standard bronze, at the works of Borbordienne, in Paris. The pose and figure reveal a man of more than average stature, slender rather than of full habit, clad in a Prince Albert coat, with open light-weight overcoat, flaps doubled back on the breast, the right hand seeking rest and seclusion below the lower buttons of the body coat, the attitude being self-confident, firm and commanding; the body sustained by strong legs, the head uncovered, the forehead high and the hair combed back. The figure will be placed in Bryant Park, New York City, before cold weather sets in, by decree of the park board. The pedestal is being built by the New England Granite Company.

---

The small-pox excitement consequent upon its prevalence in Chicago, and the possibility of its importation into Buffalo, apparently has subsided. The wise and prompt precautions taken by Dr. Ernest Wende, Health Commissioner, are commendable, and seem to have proven efficient. There is no question about the propriety of quarantine and vaccination at such times and their rigid enforcement.

---

Dr. Charles S. Hoyt, secretary of the State Board of Charities, presented a report on Immigration at the National Conference of Charities and Correction, held at Chicago, June 9, 1893. Dr. Hoyt was chairman of the committee on immigration, and his report is both interesting and exhaustive. From it we learn that the New York State Board of Charities has, since 1880, removed 1,879 alien paupers to their European homes, as follows: To England, 445; to Ireland, 405; to Scotland, 78; to Germany, 526; to Norway, 13; to Sweden, 50; to Denmark, 23; to Holland, 13; to Belgium, 1; to France, 34; to Switzerland, 68; to Italy, 91; to Austria-Hungary, 95; to Russia, 38. The whole expenditure for these removals has been $40,916.40; the expense per person, $21.78. These removals have effected a great saving to the State, and have been made without any well-founded grounds of complaint from the countries to which the persons were sent.

---

THE Cincinnati Hospital has presented its thirty-third annual report, which is for the fiscal year ending December 31, 1893. This hospital is not dependent upon charity, but receives annually an appropriation from the municipality sufficient to cover its expenses. The amount appropriated for the hospital for the year 1893 was $125,660, and the earnings from paying patients during the year were $4,102.85. The gross expenses for all purposes was $109,268.96. The hospital treated 4,954 patients at an average net cost per patient of 81.1 cents per day, or $5.67 per week. We have referred to these figures to show the proper basis on which a hospital should be managed, namely—on municipal appropriations and not the insecure and uncertain foundation of a dependency on private charity.

---

IF ALL the time, space and money expended by the medical journals in discussing, pro and con, the revision of the code of medical ethics had been devoted to the discussion of questions relating to the prevention of disease or some other equally important scientific subject, it is probable that greater progress would have been made in the science and art of medicine.

---

THE importance of membership in county medical societies is admirably set forth in an address delivered before the Allegheny (Pa.) County Bar Association, May 5, 1894, by Dr. J. C. Lange, of Pittsburg. From this admirable address, published in part in the *Pittsburg Medical Review*, we quote as follows :

> Essential to the welfare of scientific medicine are medical associations and societies ; these are numerous and noted especially for their excellent transactions and work in Allegheny county. Among the medical societies in the county, there is but one, however, which is officially representative of scientific medicine. It is the Allegheny County Medical Society. Many other societies embrace members of the County Society, or are constituted entirely of such members. Those of their members who do not also hold membership in the County Society possess no credentials as to their standing. It does not follow from this that they are ineligible and could not obtain membership ; the County Society would gladly welcome many who do not join its ranks from failure of appreciation on their part or carelessness or neglect; many who are out would be heartily appreciated in the pale of the Society. The fact remains, however, that the physician who is not a member of his County Society has no professional standing, no credentials, however well he may deserve both and however easily he might acquire them. Membership in the Allegheny County Medical Society is the only guarantee of medical respectability in the county.

